# Editorial: Whither globalization and health in an era of geopolitical uncertainty?

**DOI:** 10.1186/s12992-022-00881-x

**Published:** 2022-10-18

**Authors:** Ronald Labonté, Greg Martin, Katerini T. Storeng

**Affiliations:** 1 University of Ottawa, 600 Peter Morand Cres, K1G 5Z3 Ottawa, ON Canada; 2 Health Protection Surveillance Centre, 25-27 Gardiner Street Middle, Mountjoy, D01 A4A3 Dublin, Ireland; 3 Centre for Development and the Environment, University of Oslo, Sandakerveien 130Bygg, 20484 Oslo, Norway

**Keywords:** Globalization, COVID-19, Neoliberalism, Sustainable development goals, Governance, Climate Change, Pandemic

## Abstract

Globalization has been declared dead or dying for many years, although recently, the number of voices declaring it ‘over’ has swelled [1]. As editors of a journal interrogating how globalization affects health, we confront the question: Have the COVID-19 pandemic, Russia’s war against Ukraine, a breakdown in multilateralism, and the risk of a return to the stagflation of the 1970s finally sounded a death knell for the research and scholarship we have been publishing in the journal’s 20-year history? We think not and argue below why, in our post-pandemic fractured and fractious era, it is vitally important to retain a focus on this messy construct short-handed as ‘globalization.’

## Main text

Globalization has been declared dead or dying for many years, although recently, the number of voices declaring it ‘over’ has swelled [1]. As editors of a journal interrogating how globalization affects health, we confront the question: Have the COVID-19 pandemic, Russia’s war against Ukraine, a breakdown in multilateralism, and the risk of a return to the stagflation of the 1970s finally sounded a death knell for the research and scholarship we have been publishing in the journal’s 20-year history? We think not and argue below why, in our post-pandemic fractured and fractious era, it is vitally important to retain a focus on this messy construct short-handed as ‘globalization.’

### Globalization is dead, long live globalization

Consider, first, the contested meaning of globalization itself. Although the term only came to dominate policy discourse in the 1980s, indicative of its modern roots in the market-fundamentalism of neoliberal economics, it has a broader sociological meaning that embraces communicative, cultural, cognitive, and temporal processes [2]. As such it is not a new phenomenon but is ‘a process that has been going on for the past 5000 years’ [3], traversing eras of imperial expansion, Western colonization, rapid industrialization, emerging global governance and gradual global economic integration [4]. Neoliberalism (1980 onwards) is globalization’s most recent defining era, with impacts on health equity that have been a mainstay of much public health research.

However characterized, the processes associated with globalization (e.g., trade, movements of people, cultural exchange) are not new, but neither are they static. What the punditry declares to be globalization’s current slow demise is more indicative of another of its transformations rather than any ‘end of [globalization] history.’ The ‘hyper-globalization’ of the past four decades may be in retreat [5] as it has been since the 2008 global financial crisis and its subsequent ‘slowbalization,’ [6] marked primarily by declining growth in high- and upper-middle-income countries. Less dramatic growth slowdowns almost everywhere else suggests that, in GDP terms at least, we may have reached ‘peak [economic] globalization’.

How much of neoliberal orthodoxy will remain resiliently intact, however, is ripe for continued empirical investigation. So, too, are the promises of the International Monetary Fund and World Bank to ensure that spending on health, education, and social protection are protected in new rounds of loan conditionalities, especially given early evidence suggesting that they are not [7]. Inflation-fearing fiscal hawks, meanwhile, are sharpening their budget-slashing talons, often with the support of a growing right-wing (and alt-right) populism. As with previous rounds of structural adjustment and austerity, it will be the health of women, children, the poor, and the marginalized that will suffer most, a probable outcome that demands the sustained critical attention of global health researchers.

### Global market integration: on the wane but still globalization’s paradigmatic core

Trade has long been central to almost any globalization conceptualization, predating the 1995 birth of the World Trade Organization (WTO) and the post-2000 surge in international investment agreements. The health impacts of trade (often focusing on trade and investment agreements) continue to generate research scrutiny (https://www.biomedcentral.com/collections/trade-health). Although the impacts of aggregate trade flows on population health is mixed, there is greater consensus that the global diffusion of alcohol, tobacco, ultra-processed foods, and other unhealthy commodities enabled by trade and investment treaties poses substantial public health risk. Since trade and investment treaties are intrinsically commercial agreements negotiated by states on behalf of their corporate interests, there remains a need for independent, critical research exploring how corporate actors attempt to influence trade policy in ways detrimental to global health.

COVID-19’s sudden arrival and subsequent lockdowns created an economic shock many times greater than that of the 2008 financial crisis [8]. The first year of the pandemic saw forecasts of a sharp and persisting decline in the value of global trade, with many countries imposing import and export restrictions on goods essential to their pandemic response. Although global trade recovered more rapidly than economic models had predicted [9], some trade analysts question whether the recent era of trade openness is coming to an end. Global supply chains are considered fragile and vulnerable to any future pandemic or similar shock. Trade talk is now peppered with references to ‘re-shoring’, ‘onshoring’, ‘near-shoring,’ and ‘friend-shoring’ [10], all with an intent to promote a ‘free but secure trade [11].’ Such terms convey a cautious security-first stance that may de-risk supply, but which could further weaponize trade leading to new trade wars [5]. For many low-income countries whose economic growth of the past few decades was reliant on supplying raw or finished goods to high-income countries, a global emphasis on national security could have cascading trade-related impoverishing impacts [12], negatively and inequitably affecting public health. Conversely, an inwards economic (or regional trade economic) development emphasis could improve equitable domestic health gains if global trade, taxation, and financial market rules are amended to disproportionately benefit poorer countries. Sorting through the pathways from shifts in trade and investment rules and flows to health outcomes and their social stratification will require ongoing detailed study.

### COVID-19’s diminished but residual shadow

Wealthy nations’ failures at the height of the pandemic to agree to any meaningful waiver of the WTO TRIPS rules to improve developing countries’ access to vaccines and related health products bodes ill for future trade reforms that would improve health equity gains globally. The TRIPS waiver was only one of the global health concerns unleashed by the pandemic. Almost immediately the lack of pandemic preparedness (despite all the recent test-cases from SARS through Ebola to Zika) was glaringly obvious, as was the parlous state of health systems’ capacity in many of the world’s countries, rich and poor alike. Amidst the deluge of research and commentary articles offering short-term assessments of health system failures usually attributed to funding cuts and privatization of many health system functions [13], our journal focused on papers dealing explicitly with globalization processes affecting COVID-19, such as trade, global governance, international health law, and global health financing and partnerships. Many of these papers appear in our guest-edited special collection on ‘cross border infectious disease threats’ (https://www.biomedcentral.com/collections/cross-border-disease).

One of the researchable questions arising from COVID-19 is analyzing the global/globalization contexts (both historic and current) that might help to explain important differences between countries and their pandemic responses. There is also a need for granular studies on the effectiveness of specific government interventions in key social determinants of health (e.g., income, employment, education, housing, social protection) in reducing COVID-19-related morbidity and mortality. Such studies will be important in the ongoing development of global pandemic preparedness policies.

The pandemic’s residual shadow (apart from its knock-on political and economic impacts) is receding, with the ‘slow burn’ of antimicrobial resistance and the ‘hot burn’ of climate change re-emerging as pressing topics in global health governance. Alongside the real threat of new zoonotic outbreaks and mounting evidence of a ‘long-covid’ deterioration in many people’s health [14], health systems worldwide will face acute financing and service delivery challenges for years to come. Policy debates on what is needed to rebuild health system capacities must be informed by global health research that gives due consideration to the globalization processes that allocate the resources needed for resilient health systems. This holds true not only to mitigate future pandemic threats, but to allow countries to come even close to the health and environment targets of the Sustainable Development Goals.

### Geopolitics and the unravelling of multilateralism

The speed with which high-income countries used their central banks to put billions of dollars into advance purchase agreements for the vaccine candidates (formative research for which was largely funded by government grants in the first place) led to justifiable accusations of ‘vaccine hoarding’. It also disclosed the extent to which the multilateral era of global health cooperation was unravelling rapidly, a process that began when China, Russia (even prior to the Ukraine invasion), and recently India challenged the hegemonic presumptions of the USA. Geopolitics are always at play in anything globalization-related; but competition between states jockeying for their own sense of imperial entitlements is becoming fiercer with COVID-19 making an already tense situation appreciably worse.

Some argue that the ‘golden age’ of global solidarity for health, the first decade of the 21st century and the shift from Millennium Development to Sustainable Development Goals, is over [15]. Others contend that the WHO’s efforts to rapidly develop some form of pandemic treaty offers an opportunity for multilateral health re-engagement. Strong differences between powerful countries already exist, however, such as the USA’s initial preference for reforms to the International Health Regulations rather than a new legally binding WHO treaty, developing countries ongoing concerns with rules that could prioritize pathogen genomic sharing over therapeutic benefits sharing, and challenges in aligning any new treaty obligations with those under existing international law. There is also the suggestion that, without enforcement measures, international treaties rarely achieve their stated aims [16]. More detailed and contextualized studies of specific health-relevant global treaties and treaty-making are needed to address whether efforts expended in treaty negotiations are warranted. Surely that is a globalization question that health scholars would find hard to ignore.

### Corporate governance of the global commons

The slow evisceration of the intergovernmental institutions governing global health and its socioeconomic and environmental determinants also demands further scholarly attention. Since 2010 UN agencies have seen assessed and voluntary core funding stagnate increasing their reliance on voluntary earmarked contributions, where funders (both governmental and private) are able to control the policy and program agendas [17]. Funding for the WHO is particularly unbalanced, with less than 20% of its budget coming from assessed contributions [18], with the organization reliant on governmental and philanthropic donors and, like other UN agencies, increasingly pursuing private sector donors [19]. In a context of rising economic inequality and the concentration of wealth in transnational corporations and private individuals, it is unsurprising, if troubling, that governments and intergovernmental governing bodies are increasing their embrace of the private sector. One facet of this is the continued growth of ‘multistakeholder governance platforms,’ in which corporate representatives often have an oversized influence in what some researchers have described as ‘The Great Takeover’ [20].

As the world emerges from the peak of the pandemic to confront an increasing array of health crises (the aptly re-named ‘climate chaos’ being highest on the agenda), analyzing who holds the financial and decision-making powers in global governance becomes more critical. So, too, is enumerating what policy measures and international norms and rules are needed to ensure equitable participation of all countries, and all population groups. How the world’s peoples govern themselves is perhaps the most critical globalization issue challenging global health.

## Conclusion

Globalization is dynamic and in increasing flux. But the various ways in which it continues to impact health persist. So, too, does the need to for global health scholars to continue their rigorous interrogation of how globalization through its many processes and pathways influences health outcomes, and how those outcomes can be made more equitable and sustainable.

## Data Availability

N/A.

## Abbreviations

WTO: World Trade Organization.
TRIPS: (WTO Agreement on) Trade-Related Aspects of Intellectual Property Rights.
SARS: Severe acute respiratory syndrome.
